# Prognostic Significance of Hypophosphatemia in Kidney Transplant Patients: A Systematic Review and Meta‐Analysis

**DOI:** 10.1111/jebm.70000

**Published:** 2025-02-17

**Authors:** Nipith Charoenngam, Thanitsara Rittiphairoj, Pitchaporn Yingchoncharoen, Chalothorn Wannaphut, Thanathip Suenghataiphorn, Thitiphan Srikulmontri, Phuuwadith Wattanachayakul, Nutchapon Xanthavanich

**Affiliations:** ^1^ Division of Endocrinology Massachusetts General Hospital Harvard Medical School Boston Massachusetts USA; ^2^ Department of Medicine Faculty of Medicine, Siriraj Hospital Mahidol University Bangkok Thailand; ^3^ Department of International Health Johns Hopkins Bloomberg School of Public Health Baltimore Maryland USA; ^4^ Division of Health Systems Management Department of Community Medicine Faculty of Medicine Ramathibodi Hospital Mahidol University Bangkok Thailand; ^5^ Department of Medicine Texas Tech University Health Sciences Center Lubbock Texas USA; ^6^ Department of Medicine John A. Burns School of Medicine University of Hawai'i Honolulu Hawaiʻi USA; ^7^ Department of Internal Medicine Griffin Hospital Derby Connecticut USA; ^8^ Department of Medicine Jefferson Einstein Hospital Philadelphia Pennsylvania USA

1

Hypophosphatemia is a common electrolyte disorder in kidney transplant (KT) patients, occurring in up to 93% of cases. This condition is mediated by persistent hyperparathyroidism and elevated serum fibroblast growth factor‐23 (FGF‐23) due to long‐standing phosphate retention, leading to increased phosphaturia when the kidney's intrinsic ability to handle phosphate normalizes [1]. In severe cases, hypophosphatemia can lead to muscle weakness, cardiomyopathy, hemolytic anemia and respiratory failure [1]. However, the clinical significance of hypophosphatemia in milder cases is less well‐established. Interestingly, some studies have shown that the presence of hypophosphatemia in KT patients indicated a favorable prognosis, including decreased risks of graft failure, mortality and cardiovascular disease (CVD) [2, 3]. Other studies, however, demonstrated a U‐shaped association between serum phosphate levels and adverse outcomes [4, 5]. Therefore, using systematic review and meta‐analysis techniques, we aimed to summarize all available data to investigate the association between hypophosphatemia and various clinical outcomes, including graft failure, all‐cause mortality, cardiovascular mortality and CVD.

Three investigators (T.S., T.S., C.W.) independently searched publications indexed in PubMed and Embase databases from inception to March 27, 2024. Search terms were based on terms associated with “hypophosphatemia” and “kidney transplant.” No language restrictions were applied. The detailed search strategy is provided in Supplemental Material 1.

Eligible studies must include a cohort of KT patients with hypophosphatemia and a comparator cohort of KT patients without hypophosphatemia, excluding those with hyperphosphatemia. These studies should compare the risk of incident clinical outcomes, including all‐cause mortality, cardiovascular mortality, CVD, and graft failure. Effect estimates and 95% confidence intervals (CIs) representing the risk ratio of incident outcomes between KT patients with and without hypophosphatemia must be reported. Studies that reported the association between serum phosphate as a continuous variable and clinical outcomes were deemed ineligible, as this association may be influenced by the effect of hyperphosphatemia.

The eligibility of the retrieved articles was evaluated by three investigators (P.Y., N.X., P.W.). Any discrepancies in evaluation were resolved through discussions with the senior investigator (N.C.). The quality of each study was assessed by two investigators (N.C. and P.Y.) using the Newcastle‐Ottawa Quality Assessment Scale (NOS) for cohort studies.

Meta‐analyses were performed for outcomes reported by at least four studies. Effect estimates with standard errors were extracted from each included study. The extracted data were combined together using the generic inverse variance method as described by DerSimonian and Laird. Since the eligible studies had different study protocols and background populations, a random‐effect model was applied. The Cochran's *Q* test, complemented by the *I* ^2^, was used for assessment of statistical heterogeneity. All analyses were performed using the StataMP15.

A total of 10,907 articles were identified from the Embase and PubMed databases. Finally, 12 articles [2, 3, 4, 5, 6, 7, 8, 9, 10, 11, 12, 13] met the eligibility criteria, all of which were full‐length articles (Figure S1). Among these, the associations between hypophosphatemia and clinical outcomes were reported in 10 studies for graft failure [2–7, 9–11, 13], 10 studies for all‐cause mortality [2–5, 8–13], 3 studies for cardiovascular mortality [3, 6, 13], and 2 studies for CVD [3, 10]. One study was conducted in pediatric patients [7]. Of note, the patients studied by Aarts et al. [6] and Van Londen et al. [13] were subsets of those in the van der Plas et al. study [5], and the patients studied by Kim et al. [9] were subsets of those in the Jeon et al. [4] study. These studies [6, 9, 13] were therefore excluded from the meta‐analyses of graft failure and all‐cause mortality. Consequently, seven studies [2–5, 7, 10, 11] were included in the meta‐analysis of graft failure and eight studies [2–5, 8, 10–12] were included for the meta‐analysis of all‐cause mortality. All except for two studies [2, 3] were determined to be of high quality with a NOS score of at least 7. The characteristics of the studies included in the meta‐analysis are shown in Table S1.

The meta‐analysis of eight studies revealed that hypophosphatemia was associated with a nonsignificant decreased risk of all‐cause mortality with a pooled hazard ratio (HR) of 0.86 (95% CI 0.61 to 1.20; *I* ^2^ = 65.2%, *p* = 0.005) (Figure 1A). The meta‐analysis of seven studies revealed that hypophosphatemia was associated with a nonsignificant increased risk of graft failure with a pooled HR of 1.08 (95% CI 0.56 to 2.09; *I* ^2^ = 91.0%, *p* < 0.001) (Figure 1B). The funnel plots and Egger's regression test for both analyses were not suggestive of publication bias (Figure S2). Since estimated glomerular filtration rate (eGFR) was considered a potential confounder, a sensitivity analysis was performed by excluding the three studies (3, 10, 12) that did not report effect estimates adjusted for eGFR. The pooled HR for the risk of all‐cause mortality slightly decreased from the original result to 0.79 (95% CI 0.49 to 1.27, Figure S3A), while the pooled HR for the risk of graft failure slightly increased to 1.17 (95% CI 0.53 to 2.59, Figure S3B).

**FIGURE 1:**
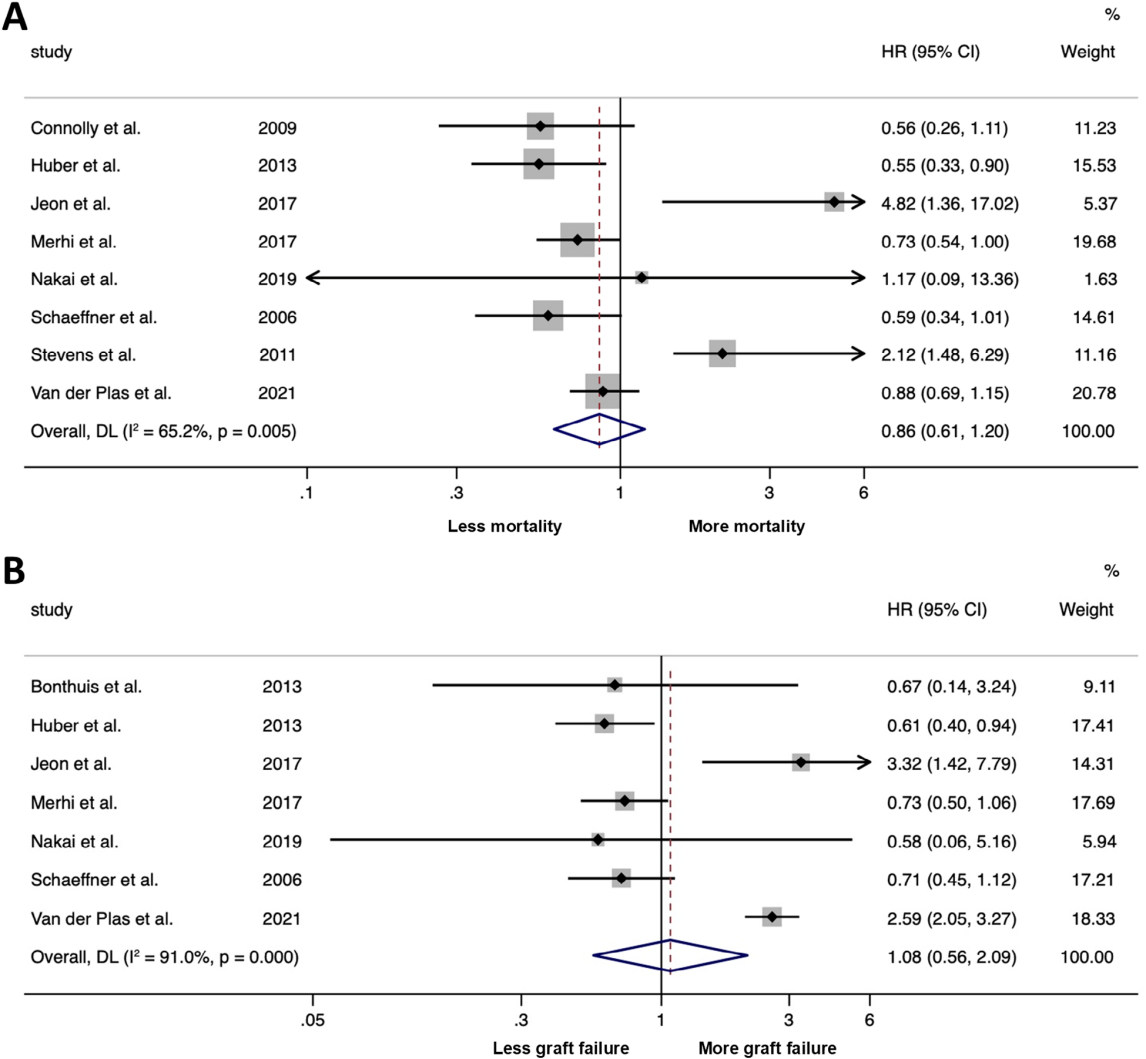
Forest plot of the meta‐analysis of the association between hypophosphatemia and risk of all‐cause mortality (A) and graft failure (B) among kidney transplant patients. DL, DerSimonian‐Laird random‐effects model; HR, hazard ratio; CI, confidence interval.

For cardiovascular mortality, Aarts et al. [6] reported a statistically significant association between mild hypophosphatemia (serum phosphorus 1.55– 2.17 mg/dL) and severe hypophosphatemia (serum phosphorus <1.55 mg/dL) and a decreased risk of cardiovascular mortality with hazard ratios of 0.25 (95% CI 0.11 to 0.58) and 0.29 (95% CI 0.13 to 0.67), respectively. Utilizing the same patient data, Van Londen et al. [13] reported that mild and severe hypophosphatemia were associated with a decreased risk of cardiovascular mortality with hazard ratios of 0.45 (95%CI 0.21 to 0.95) and 0.38 (95%CI 0.17 to 0.84), after adjustments for confounders. Merhi et al. [3] reported a trend toward a lower incidence of cardiovascular mortality among patients with serum phosphorus in the lowest serum phosphorus quartile (serum phosphorus <2.51 mg/dL, incidence 7.0 per 1000 person‐years) compared with those in the second quartile (serum phosphorus 2.52– 2.90 mg/dL, incidence 7.9 per 1000 person‐years) and third quartile (serum phosphorus 2.91– 3.22 mg/dL, 11.4 per 1000 person‐years). A similar trend was observed by Merhi et al. [3] for the outcome of incident CVD, with 26.8 per 1000 person‐years in the lowest serum phosphorus quartile compared with the second and third quartiles of 31.8 and 40.4 per 1000 person‐years, respectively. In contrast, Nakai et al. [10] reported a nonsignificant difference in the rates of CVD, with 0% among patients with hypophosphatemia at 12 months.

This is the first systematic review and meta‐analysis investigating the prognostic significance of hypophosphatemia in KT patients. The meta‐analysis revealed a trend toward decreased all‐cause mortality (pooled HR = 0.86) in KT patients with hypophosphatemia, although not reaching statistical significance. However, no association between hypophosphatemia and graft failure was observed. In addition, hypophosphatemia may be associated with decreased risks of cardiovascular mortality and CVD based on limited evidence. These findings provide additional insights into the impact of phosphate homeostasis in KT. Additionally, it is crucial to note that there was a marked discrepancy in the results of the studies included in our meta‐analysis. While most studies showed an inverse association between hypophosphatemia and graft failure and all‐cause mortality [2, 3, 8, 11], some studies showed the opposite association [4, 5] and few showed a null association [7, 10].

There are mechanisms supporting both the protective and detrimental roles of hypophosphatemia on cardiovascular, renal, and mortality outcomes. On one hand, the presence of hypophosphatemia may indicate healthy transplanted kidneys, as it suggests the kidney's intrinsic ability to excrete phosphate. On the other hand, hypophosphatemia may indicate a persistent elevation of FGF‐23, a hormone known to be a cardiovascular toxin that promotes cardiac hypertrophy and is associated with increased risks of CVD and mortality in multiple clinical studies [14]. Additionally, hypophosphatemia may be a marker of poor nutritional status, including low protein intake, vitamin D deficiency, and other micronutrient deficiencies, all of which are associated with impaired physical conditions and increased mortality risks [15].

Other possible explanations for the heterogeneity of the findings include differences in patient characteristics and study design. Notably, the variables adjusted in multivariate analysis may influence the reported association. For example, in a study by Van der Plas et al. [5], hypophosphatemia was statistically significantly associated with a decreased risk of graft failure after adjustment for age and sex, with a hazard ratio of 0.43 (95% CI 0.25 to 0.74). However, the association reversed (HR = 2.17, 95% CI 1.24 to 3.78) after adding eGFR and other variables to the model. This suggests that eGFR confounded the association between hypophosphatemia and graft failure.

This study has certain limitations that should be noted. First, the limited number of studies on cardiovascular mortality and cardiovascular outcomes prohibited performing a meta‐analysis. Further studies are required to increase the confidence in the findings. Second, the cut‐off values for hypophosphatemia and the timing of serum phosphate measurements were not standardized across the studies, which limits the reliability of the meta‐analysis. Additionally, it remains unknown whether hypophosphatemia at different times after KT has different clinical significance, and whether phosphate repletion would modify the outcomes. These questions require further investigation. Finally, the small number of studies included in the meta‐analysis could jeopardize the validity and interpretation of the funnel plots.

In conclusion, the present systematic review and meta‐analysis explore the prognostic significance of hypophosphatemia in KT patients. The meta‐analysis indicated a nonsignificant trend towards reduced all‐cause mortality in KT patients with hypophosphatemia. No association between hypophosphatemia and graft failure was observed. However, limited evidence suggests that hypophosphatemia may be associated with lower risks of cardiovascular mortality and CVD. It is important to highlight the substantial variability in the outcomes of the included studies. While most studies reported an inverse relationship between hypophosphatemia and both graft failure and all‐cause mortality, some studies found the opposite association, which is possibly due to differences in patient characteristics and study design.

## Conflicts of Interest

The authors declare no conflicts of interest.

## Supporting information



Supporting Information
