# Supplementary material for: Prognostic Significance of Hypophosphatemia in Kidney Transplant Patients: A Systematic Review and Meta‐Analysis
Source: J Evid Based Med. 2025 Feb 17;18(1):e70000. doi: 10.1111/jebm.70000 (PMC11831411; doi:10.1111/jebm.70000)
Supplement: Supplementary file 1 — Supporting Information [file JEBM-18-0-s001.docx]

**Supplemental Material**

Supplemental Material 1. Search strategy

**PubMed**

("renal transplant"[Title/Abstract] OR "kidney transplant"[Title/Abstract] OR "renal transplantation"[Title/Abstract] OR "kidney transplantation"[Title/Abstract] OR "kidney graft"[Title/Abstract]) AND ("phosphate"[Title/Abstract] OR "phosphorus"[Title/Abstract] OR "hypophosphatemia"[Title/Abstract] OR "hypophosphataemia"[Title/Abstract])

**Embase**

1. 'kidney transplantation'/exp OR 'kidney transplantation'
2. 'kidney graft'/exp OR 'kidney graft'
3. ('kidney'/exp OR kidney) AND ('transplant'/exp OR transplant)
4. 'renal transplant recipient'/exp OR 'renal transplant recipient'
5. ('renal'/exp OR renal) AND ('transplant'/exp OR transplant)
6. ('renal'/exp OR renal) AND ('transplantation'/exp OR transplantation)
7. 'phosphate'/exp OR 'phosphate'
8. 'hypophosphatemia'/exp OR 'hypophosphatemia'
9. 'hypophosphataemia'/exp OR hypophosphataemia
10. 'phosphorus'/exp OR 'phosphorus'
11. #1 OR #2 OR #3 OR #4 OR #5 OR #6
12. #7 OR #8 OR #9 OR #10
13. #11 AND #12

Supplementary Figure 1. Study identification and literature review process

**
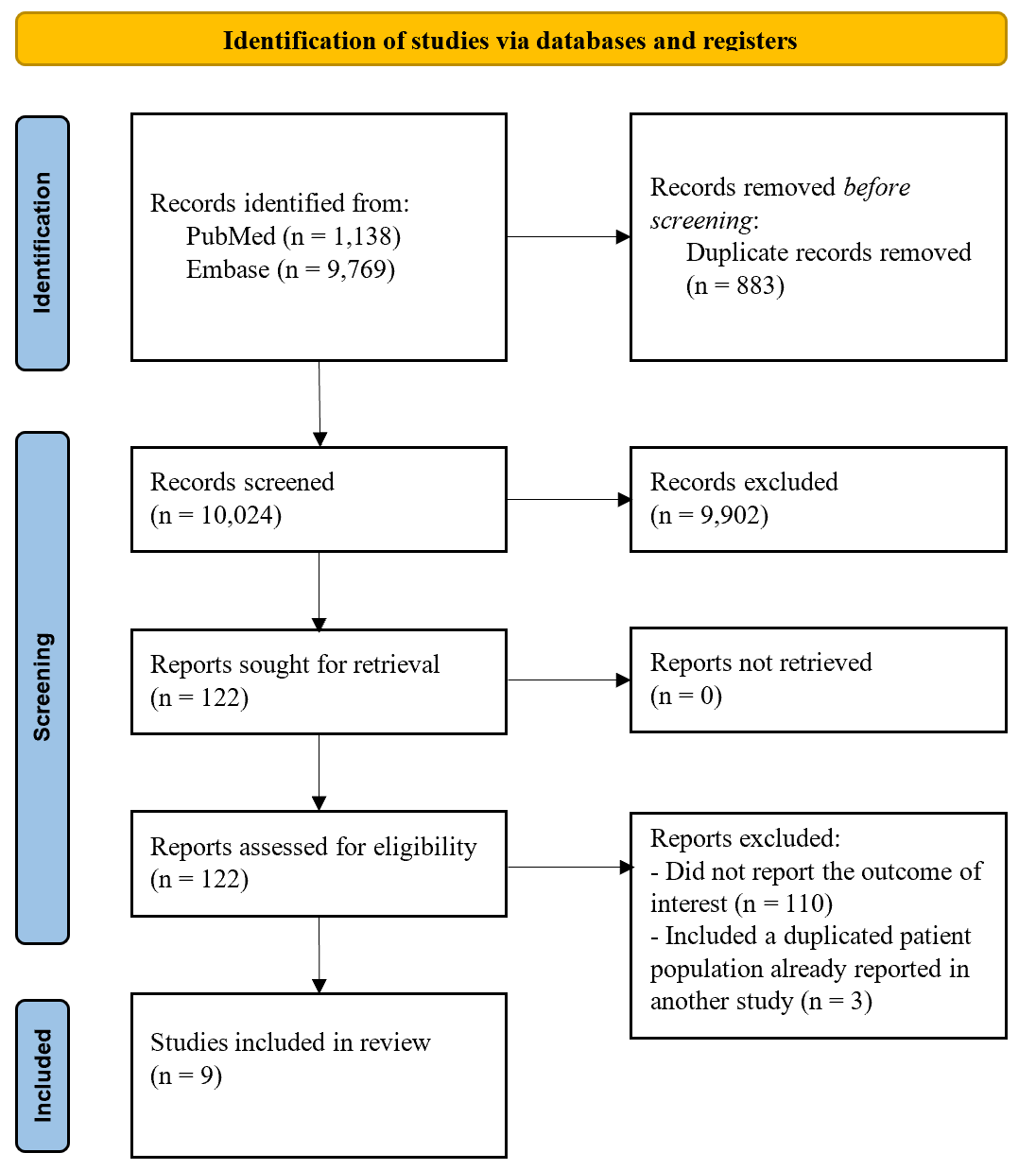
**

Supplementary Figure 2. Funnel plot of the meta-analysis of the association between hypophosphatemia and risk of all-cause mortality (A) and graft failure (B) among kidney transplant patients. SE: standard error; HR: hazard ratio.


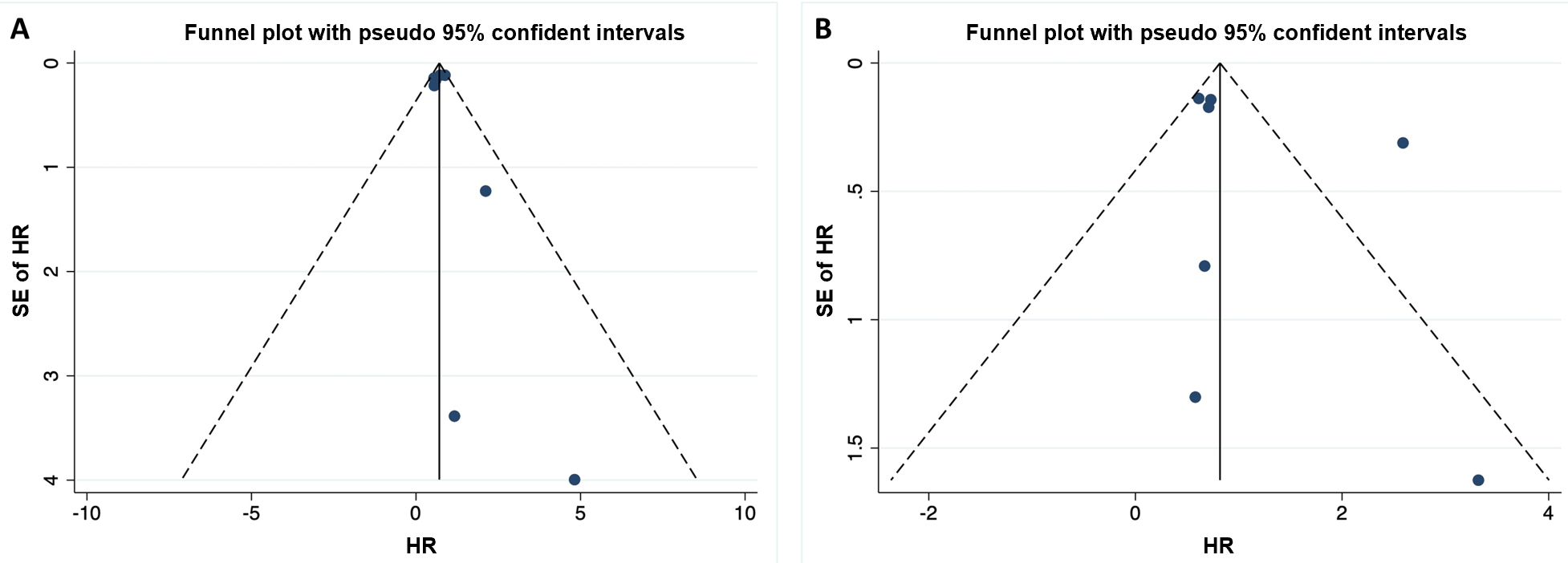


Egger’s regression test p-value: 0.438 (A), 0.832 (B).

**Supplementary Figure 3. Forest plot of the sensitivity meta-analysis of the association between hypophosphatemia and risk of all-cause mortality (A) and graft failure (B) among kidney transplant patients, excluding studies that did not report effect estimates adjusted for estimated glomerular filtration rate.** DL: DerSimonian-Laird random-effects model; HR: hazard ratio; CI: confidence interval.


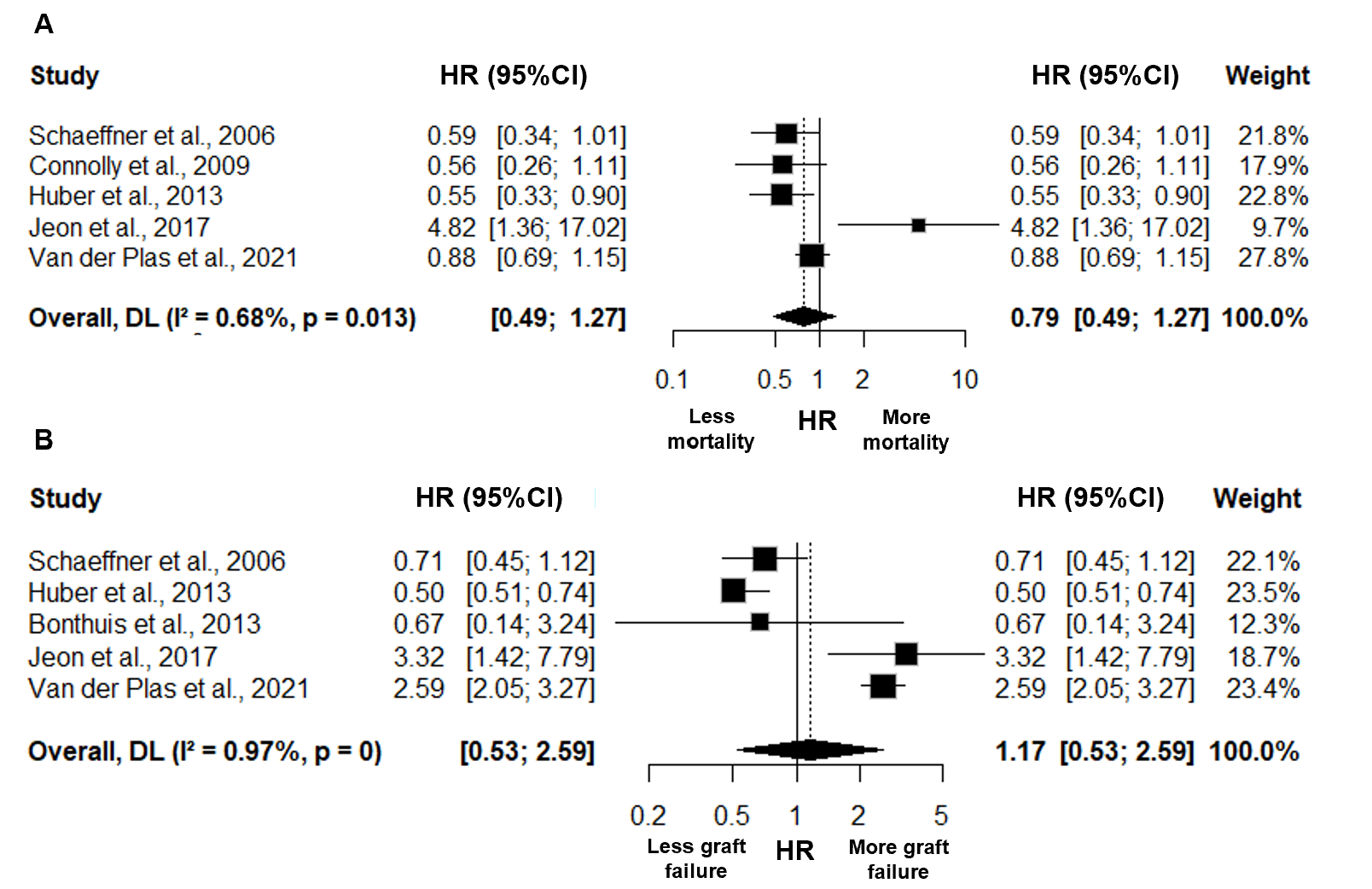


Supplementary Table 1. Main characteristics of the cohort studies investigating clinical outcomes associated with hypophosphatemia in kidney transplant patients included in the meta-analysis

| Characteristics | Schaeffner et al.^1^ | | Conolly et al.^2^ | Stevens et al. ^3^ | Huber et al. ^4^ | Bonthuis et al. ^5^ | Jeon et al. ^6^ | Merhi et al. ^7^ | Nakai et al. ^8^ | van der Plas et al. ^9^ |
| --- | --- | --- | --- | --- | --- | --- | --- | --- | --- | --- |
| Country | Austria | | Ireland | Scotland | Germany | European countries | South Korea | USA | Japan | The Netherlands |
| Year of publication | 2006 | | 2009 | 2011 | 2013 | 2015 | 2017 | 2017 | 2019 | 2020 |
| Number of patients | 708 | | 379 | 377 | 776 | 1,237 | 2,786 | 3,138 | 90 | 2,769 |
| Recruitment of patients | KT patients were recruited from the transplant clinic of the Vienna General Hospital during 1996 – 1998. | | KT patients were recruited from renal transplant clinics in two hospitals in Northern Ireland during June 2000 – December 2002. | KT patients who underwent a first deceased donor renal transplant during January 1999 – December 2008 were identified from the medical record of the Renal Transplant Unit at the Western Infirmary, Glasgow | Adult patients who underwent KT during 1 January 2000 to 31 December 2010 were recruited. | Pediatric KT patients were identified from the ESPN/ERA-EDTA registry, which include data from the following countries and periods: Belarus (2009-2010), Belgium (2010-2011), Denmark (2006-2011), Finland (2000-2011), Hungary (2010), Norway (2009-2011), Portugal (2008-2011), Slovenia (2007-2011), Turkey (2011-2012), and the UK (2002-2012). | Patients with recorded serum phosphorus levels at 1 year after KT at Seoul National University Hospital, Boramae Medical Center, and Asan Medical Center during January 1997 – August 2012 were recruited. | KT patients who attended the FAVORIT study (NCT00064753) and had complete data were selected for analysis. | Patients who underwent KT at Red Cross Fukuoka Hospital during 2008 – 2016 were recruited. | Patients who underwent KT at the University Medical Center Groningen during March 1970 – January 2016 were recruited. |
| Definition of patients with hypophosphatemia | KT patients with serum phosphorus in the lowest quintile (<2.60 mg/dL) | | KT patients with serum phosphorus in the lowest tertile (<2.85 mg/dL) | KT patients with serum phosphorus <2.79 mg/dL at 1 year after transplant | KT patients with serum phosphorus <2.48 mg/dL at 6 months after transplant | KT patients with serum phosphorus below the EPDWG target | KT patients with serum phosphorus <2.5 mg/dL | KT patients with serum phosphorus in the 1^st^ quartile (<2.51 mg/dL) | KT patients with serum phosphorus in the 1^st^ quartile (<2.7 mg/dL) at 12 months after transplant | KT patients with serum phosphorus <2.17 mg/dL |
| Definition of comparators | KT patients with serum phosphorus in the 3^rd^ quintile (3.00 – 3.34 mg/dL) | | KT patients with serum phosphorus in the middle tertile (2.85 – 3.47 mg/dL) | KT patients with serum phosphorus 2.79 – 3.44 mg/dL | KT patients with serum phosphorus 2.48 – 4.65 mg/dL at 6 months | KT patients with serum phosphorus within the EPDWG target | KT patients with serum phosphorus 3.5 – 3.99 mg/dL | KT patients with serum phosphorus in the 2^nd^ and 3^rd^ quartile (2.52 – 3.22 mg/dL) | The rest of the studied patients | KT patients with serum phosphorus 2.17 – 4.64 mg/dL |
| Clinical outcomes | All-cause mortality; overall graft failure | | All-cause mortality | All-cause mortality | All-cause mortality; death-censored graft failure | Overall graft failure | All-cause mortality; death-censored graft failure | All-cause mortality; death-censored graft failure; cardiovascular mortality; cardiovascular disease | All-cause mortality; overall graft failure; cardiovascular disease | All-cause mortality; graft failure |
| Average duration of follow-up (years) | 6.1 | 6.1 | | 4.9 | N/A | 3.0 | 6.5 | 4.0 | 4.8 | 16.3 |
| Average age of patients (years) | 52.2 | 47.3 | | 45.8 | 48.5 | N/A | 41.7 | 51.6 | 47.0 | 47.0 |
| Percentage of female | 39.9% | 35.9% | | 37.7% | 38.4% | 40.1% | 40.7% | 37.1% | 28.9% | 42.3% |
| Participants’ underlying renal disease | DN 6.6%; GN 34.2%; interstitial nephritis 16.1%; PKD 13.7%; other specified conditions 8.3%; unknown 21.1%. | N/A | | N/A | N/A | Congenital anomaly 44.4%; GN 9.2%; cystic kidneys 10.4%; hereditary nephropathy 14.7%; ischemic renal failure 2.1%; HUS 2.9%; metabolic disorders 3.1%; vasculitis 1.1%; others 12.1% | N/A | N/A | N/A | GN 21.6%, interstitial nephritis 12.1%, cystic kidney disease 12.1%, congenital kidney disease 4.6%, renal vascular disease 9.7%, DN 7.0%, others 6.7%, unknown 20.3%, missing 0.4% |
| Transplant type | LD 4.7%; DD 95.3% | N/A | | DD 100% | N/A | LD 36.7%; DD55.6%; unknown 7.7% | LD 73.5%; DD 26.5% | LD 43.2%; DD 56.8% | LD 78.9%; DD 21.1% | LD 28.4%; DD 78.5%; unknown 0.1% |
| Variables adjusted in multivariate analysis | Age, sex, eGFR, CRP, plasma homocysteine, BMI, DN, donor sex, time from first RRT to KT | Age, sex, smoking, diabetes, SBP, total cholesterol, HDL, eGFR, hsCRP | | N/A | Analysis of all-cause mortality: none Analysis of graft failure: donor type, donor age, eGFR at 6 months | Age, sex, eGFR, and year of transplantation | Age, sex, donor type, DM, BMI, acute rejection, eGFR, serum albumin, hemoglobin and total cholesterol at 1 year after KT | N/A | N/A | Age, sex, time-updated eGFR, proteinuria, donor age, donor sex, donor status, ischemia time, number of HLA mismatches, primary renal disease, CMV infection, antihypertensive drug use, SBP. Calcium, dialysis vintage, decade of transplantation, immunosuppressive drug use, delayed graft function |
| Newcastle-Ottawa score | Selection: 4  Comparability: 2  Outcome: 3 | Selection: 4  Comparability: 2  Outcome: 3 | | Selection: 4  Comparability: 0  Outcome: 3 | Selection: 3  Comparability: 1  Outcome: 2 | Selection: 4  Comparability: 2  Outcome: 3 | Selection: 4  Comparability: 2  Outcome: 3 | Selection: 3  Comparability: 0  Outcome: 3 | Selection: 4  Comparability: 0  Outcome: 3 | Selection: 4  Comparability: 2  Outcome: 3 |

BMI: body mass index; CMV: cytomegalovirus; CRP: C-reactive protein; DD: deceased donor; DM: diabetes mellitus; DN: diabetic nephropathy; eGFR: estimated glomerular filtration rate; EPDWG: european pediatric dialysis working group; ESPN/ERA-EDTA: european society for pediatric nephrology/european renal association-european dialysis and transplant association; GN: glomerulonephritis; HLA: human leukocyte antigen; hsCRP: high-sensitivity c-reactive protein; HUS: hemolytic-uremic syndrome; KT: kidney transplant; LD: living donor; N/A: not applicable; PKD: polycystic kidney disease; RRT: renal replacement therapy; SBP: systolic blood pressure; UK: United Kingdom; USA: United States of America.

**REFERENCES**

1. Schaeffner ES, Födinger M, Kramar R, Sunder-Plassmann G, Winkelmayer WC. Prognostic associations of serum calcium, phosphate and calcium phosphate concentration product with outcomes in kidney transplant recipients. *Transpl Int*. 2007;20(3):247-55.

2. Connolly GM, Cunningham R, McNamee PT, Young IS, Maxwell AP. Elevated serum phosphate predicts mortality in renal transplant recipients. *Transplantation*. 2009;87(7):1040-4.

3. Stevens KK, Morgan IR, Patel RK, Geddes CC, Mark PB, Jardine AG, et al. Serum phosphate and outcome at one year after deceased donor renal transplantation. *Clin Transplant*. 2011;25(2):E199-204.

4. Huber L, Naik M, Budde K. Frequency and long-term outcomes of post-transplant hypophosphatemia after kidney transplantation. *Transpl Int*. 2013;26(10):e94-6.

5. Bonthuis M, Busutti M, van Stralen KJ, Jager KJ, Baiko S, Bakkaloğlu S, et al. Mineral metabolism in European children living with a renal transplant: A European society for paediatric nephrology/European renal association–european dialysis and transplant association registry study. *Clin J Am Soc Nephrol*. 2015;10(5):767-75.

6. Jeon HJ, Kim YC, Park S, Kim CT, Ha J, Han DJ, et al. Association of Serum Phosphorus Concentration with Mortality and Graft Failure among Kidney Transplant Recipients. *Clin J Am Soc Nephrol*. 2017;12(4):653-62.

7. Merhi B, Shireman T, Carpenter M, Kusek J, Jacques P, Pfeffer M, et al. Serum phosphorus and cardiovascular disease outcomes, graft failure, or total mortality in chronic kidney transplant recipients. *Am J Transplant*. 2017;17:760.

8. Nakai K, Mitsuiki K, Kuroki Y, Nishiki T, Motoyama K, Nakano T, et al. Relative hypophosphatemia early after transplantation is a predictor of good kidney graft function. *Clin Exp Nephrol*. 2019;23(9):1161-8.

9. van der Plas WY, Gomes Neto AW, Berger SP, Pol RA, Kruijff S, Bakker SJL, et al. Association of time-updated plasma calcium and phosphate with graft and patient outcomes after kidney transplantation. *Am J Transplant*. 2021;21(7):2437-47.
